# Decreased orbital fat and enophthalmos due to bimatoprost: Quantitative analysis using magnetic resonance imaging

**DOI:** 10.1371/journal.pone.0214065

**Published:** 2019-03-27

**Authors:** Tomoaki Higashiyama, Takayuki Minamikawa, Masashi Kakinoki, Osamu Sawada, Masahito Ohji

**Affiliations:** Department of Ophthalmology, Shiga University of Medical Science, Otsu, Shiga, Japan; Massachusetts Eye & Ear Infirmary, Harvard Medical School, UNITED STATES

## Abstract

We quantitatively determined the relation between the decrease in orbital fat and enophthalmos due to bimatoprost using magnetic resonance imaging (MRI). Nine orbits in nine patients were treated unilaterally with bimatoprost for glaucoma or ocular hypertension. The contralateral orbits were used as controls. The volumes of the orbital tissues and the enophthalmos were measured using MRI. The mean volumes on the treated and untreated sides were, respectively, 14.6 ± 2.1 and 17.0 ± 4.3 cm^3^ for orbital fat (*P* = 0.04) and 3.4 ± 0.5 and 3.3 ± 0.5 cm^3^ for total extraocular muscles (*P* = 0.85). The mean enophthalmos values were 14.7 ± 2.5 and 16.0 ± 2.3 mm on the treated and untreated sides, respectively (*P* = 0.002). The data acquired by quantitatively measuring the volumes of orbital fat and enophthalmos on MRI showed that each might be reduced by bimatoprost administration. The enophthalmos could be caused by the bimatoprost-induced decrease in orbital fat.

## Introduction

Glaucoma is a disease with chronic progression of visual field defects [1]. Previous studies reported that intraocular pressure (IOP) reduction helps prevent the progression of glaucoma [2–6]. Prostaglandin (PG) F_2α_ analogs have been effective and widely used in patients with glaucoma and ocular hypertension [7–9]. These PGF_2α_ analogs have been approved as first-line treatment for glaucoma because of their potent IOP reduction activity, which, when administered in the morning, is maintained throughout the day [10–12].

Clinically, PGF_2α_ analogs have few negative systemic side effects [13]. They do have some local side effects, however, such as causing prostaglandin-associated periorbitopathy (PAP), including periorbital fat atrophy, enophthalmos, deepening of the upper eyelid sulcus (DUES), and upper eyelid ptosis [14–19]. The mechanism of the symptoms has not been clearly identified but is thought to be related to the effect of PGF_2α_ on adipocytes [20]. PAP has been reported to be more frequently associated with bimatoprost administration than with that of other PGF_2α_ analogs, such as latanoprost, travoprost, and tafluprost [21, 22].

Although enophthalmos seems to be caused by the PGF_2α_ analog-associated decrease in orbital fat, the mechanism has not been shown quantitatively by measuring the orbital fat volume. The purpose of this study was to use magnetic resonance imaging (MRI) to reveal quantitatively the relation between the bimatoprost-associated decrease in orbital fat and the appearance of enophthalmos.

## Materials and methods

### Subjects

This observational cross-sectional study was approved by the institutional review board of Shiga University of Medical Science. The study adhered to the tenets of the Declaration of Helsinki. Written informed consent was obtained from each patient in the study.

We studied nine orbits of nine patients (mean ± SD age 62.9 ± 16.0, range 34–80 years) who were treated unilaterally with bimatoprost (Allergan, Inc., Irvine, CA, USA) for glaucoma or ocular hypertension for more than 6 months (mean 29.0 ± 14.2 months, range 11–56 months) in our Department of Ophthalmology between November 2015 and March 2018. The patients’ nine contralateral orbits were used as controls. The characteristics of the patients are shown in Table 1.

**Table 1:** Characteristics of patients.

Case	Age	Sex	Disease	Eye with disease	Duration of the bimatoprost treatment (months)
1	75	M	Primary angle-closure glaucoma	Right	11
2	56	M	Exfoliation glaucoma	Right	32
3	57	M	Exfoliation glaucoma	Left	14
4	80	F	Secondary open angle glaucoma due to uveitis	Right	28
5	34	F	Steroid-induced glaucoma	Right	28
6	75	M	Neovascular glaucoma	Right	56
7	68	M	Neovascular glaucoma	Right	28
8	41	M	Exfoliation glaucoma	Left	48
9	80	M	Exfoliation glaucoma	Right	16

F-female, M-male

None of the patients had a history of treatment with PGF_2α_ analogs for the contralateral eye, prior extraocular surgery including scleral buckling and encircling procedures, an orbital disease such as trauma, an inflammatory disease of unknown origin in the orbit, or for whom MRI examination posed a risk.

### Volume measurements

All patients were examined using a 3.0-tesla MRI unit (Signa HDxt 3.0 T; GE Healthcare, Little Chalfont, UK) at Shiga University of Medical Science Hospital. Coronal, axial, and sagittal MRI images using T2-weighted spin echo were used to measure the volumes of each tissue in the orbit in a manner similar to that described in previous studies [23, 24]. The slice thickness of the images was 1.5 mm.

**Fig 1:**
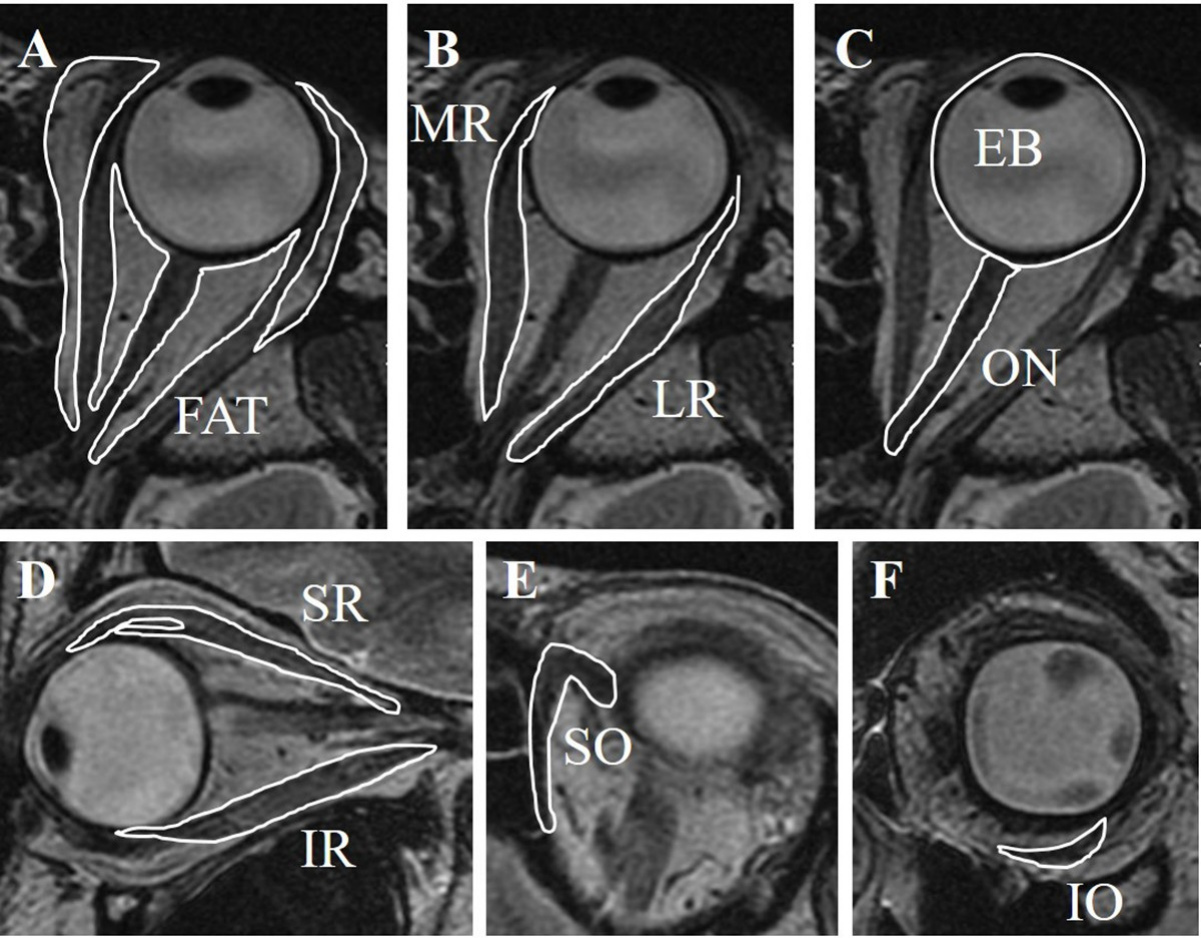
Volume measurements. Cross-sectional areas of each tissue were measured. The volumes of the tissues were calculated by multiplying the sum of the cross-sectional areas by the slice increment (1.5 mm). **A**, Orbital fat. **B**, Lateral rectus (LR) and medial rectus (MR). **C**, Optic nerve (ON) and eyeball (EB). **D**, Superior rectus (SR) and inferior rectus (IR). **E**, Superior oblique. **F**, Inferior oblique (IO).

The cross-sectional areas (CSAs) of the orbital fat, extraocular muscles, optic nerve, and eyeball were measured by tracing outlines of the tissue on the images using Aquarius iNtuition software (TeraRecon, San Mateo, Foster City, CA, USA) (Fig 1). CSAs on axial images were used for the orbital fat, lateral rectus, medial rectus, superior oblique, eyeball, and optic nerve measurements. CSAs on sagittal images were used for the superior rectus and inferior rectus measurements. CSAs on coronal images were used for the inferior oblique measurements. The orbital fat was traced together with the lacrimal gland because it was difficult to separate them on the MRI images. The superior rectus was traced along with the levator palpebrae muscle for the same reason. The volumes of the tissues were calculated by multiplying the sum of the CSAs × the slice increment. The volumes of the images were measured in a masked fashion by one technician (F.K.).

### Enophthalmos measurements

**Fig 2:**
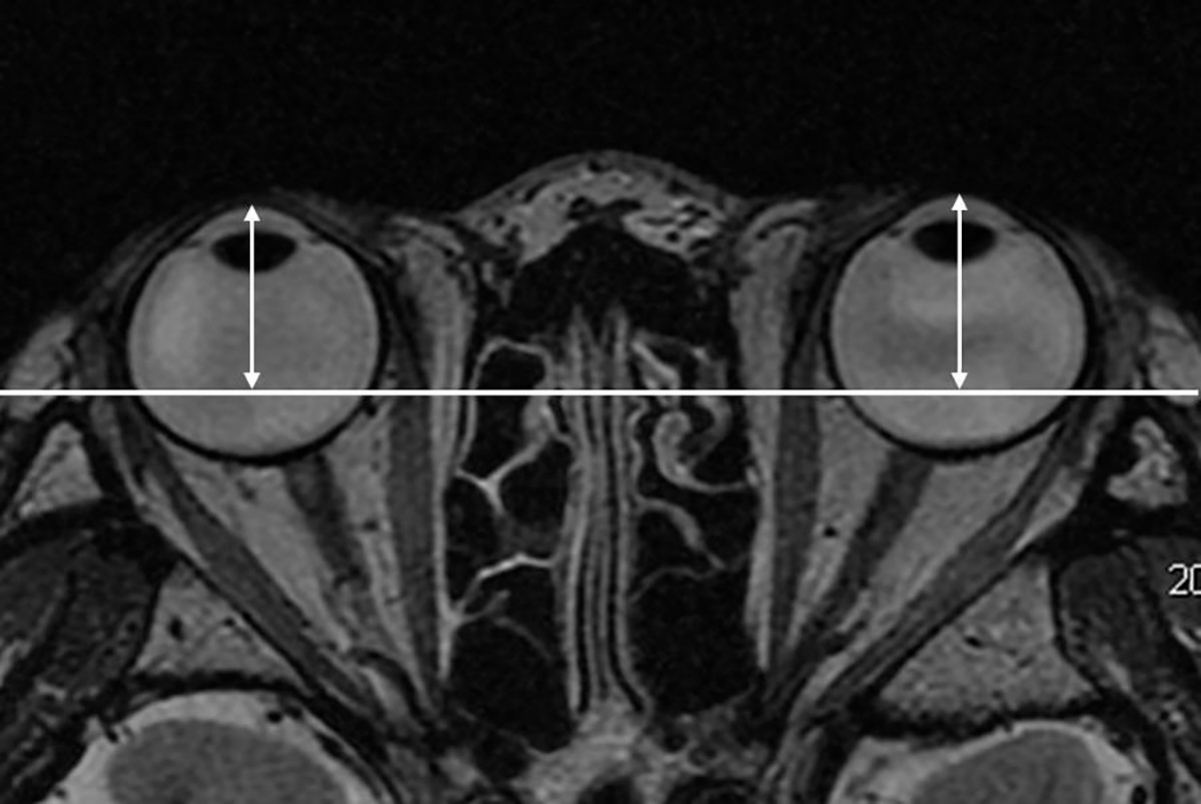
Enophthalmos measurements. A baseline was demarcated between the bilateral frontal processes of the zygomatic bones. The perpendicular distance from the top point of the corneal surface to the baseline was defined as the enophthalmos value.

The enophthalmos was measured on axial images using Aquarius iNtuition software (TeraRecon: https://www.terarecon.com) in a manner similar to that used in previous studies [24]. A baseline was demarcated between the bilateral frontal processes of the zygomatic bones. The perpendicular distance from the top point of the corneal surface to the baseline was defined as the enophthalmos value (Fig 2). Enophthalmos values were also measured in a masked fashion by one technician (F.K.).

### Statistical analysis

All statistical analyses were performed using SPSS Statistics 22 software (IBM, Armonk, NY, USA). The normality of the numerical variables was evaluated using the Shapiro–Wilk test. A paired *t*-test was used to compare volumes and values between the orbits treated with bimatoprost and their untreated contralateral orbits. Pearson’s product–moment correlation coefficient was used to analyze the correlations. The values are expressed as means ± SD. A value of *P* < 0.05 indicated statistical significance.

## Results

### Volumes

The mean volumes on the treated and untreated sides are shown in Table 2.

**Table 2:** Mean orbital tissue volumes and enophthalmos values.

	The treated side	The untreated side	P-value
Orbital fat (cm^3^)	14.6 ± 2.1	17.0 ± 4.3	0.04
Total extraocular muscles (cm^3^)	3.4 ± 0.5	3.3 ± 0.5	0.85
Eyeball (cm^3^)	9.3 ± 1.2	9.3 ± 1.0	0.85
Optic nerve (cm^3^)	0.4 ± 0.1	0.5 ± 0.1	0.10
Enophthalmos (mm)	14.7 ± 2.5	16.0 ± 2.3	0.002

All values are shown as means ± standard deviation.

**Fig 3:**
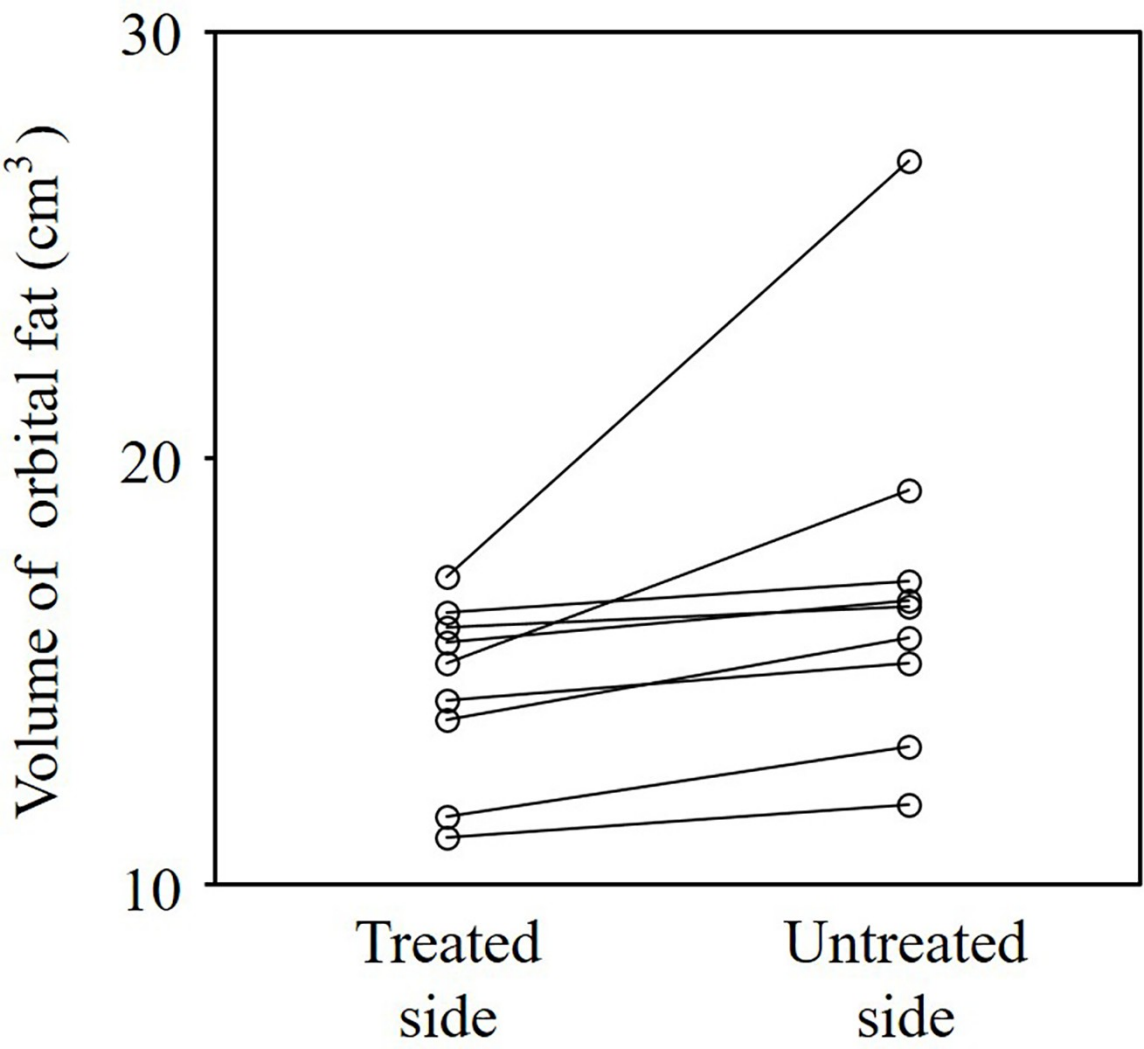
Orbital fat volumes on the treated and untreated sides. The mean orbital fat volume was significantly smaller on the treated side than on the untreated side (*P* = 0.04). The volumes of the orbital fat on the treated side were smaller than those on the untreated side in all patients, although the differences were slight in some patients.

**Fig 4:**
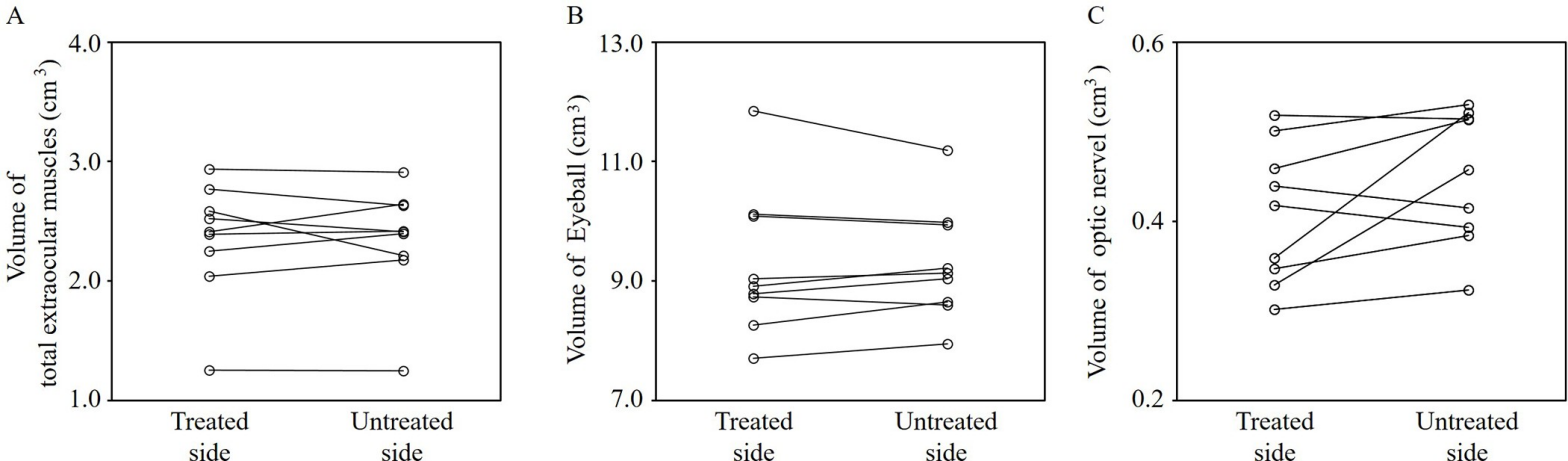
Orbital tissues’ volumes on the treated and untreated sides. **A**, Total extraocular muscles. **B**, Eyeball. **C**, Optic nerve. There were no significant differences in the mean volumes between the two orbits (total extraocular muscles *P* = 0.85; eyeball *P* = 0.85; optic nerve *P* = 0.10).

The mean volume of orbital fat on the treated side was significantly smaller than that on the untreated side (*P* = 0.04) (Fig 3). In all patients, the volume of orbital fat was smaller on the treated side than on the untreated side, although the differences were minimal in some patients. There were no significant differences in the mean volumes between the two orbits for the extraocular muscles, eyeballs, or optic nerve (total extraocular muscles *P* = 0.85; eyeball *P* = 0.85; optic nerve *P* = 0.10) (Fig 4).

### Enophthalmos

**Fig 5:**
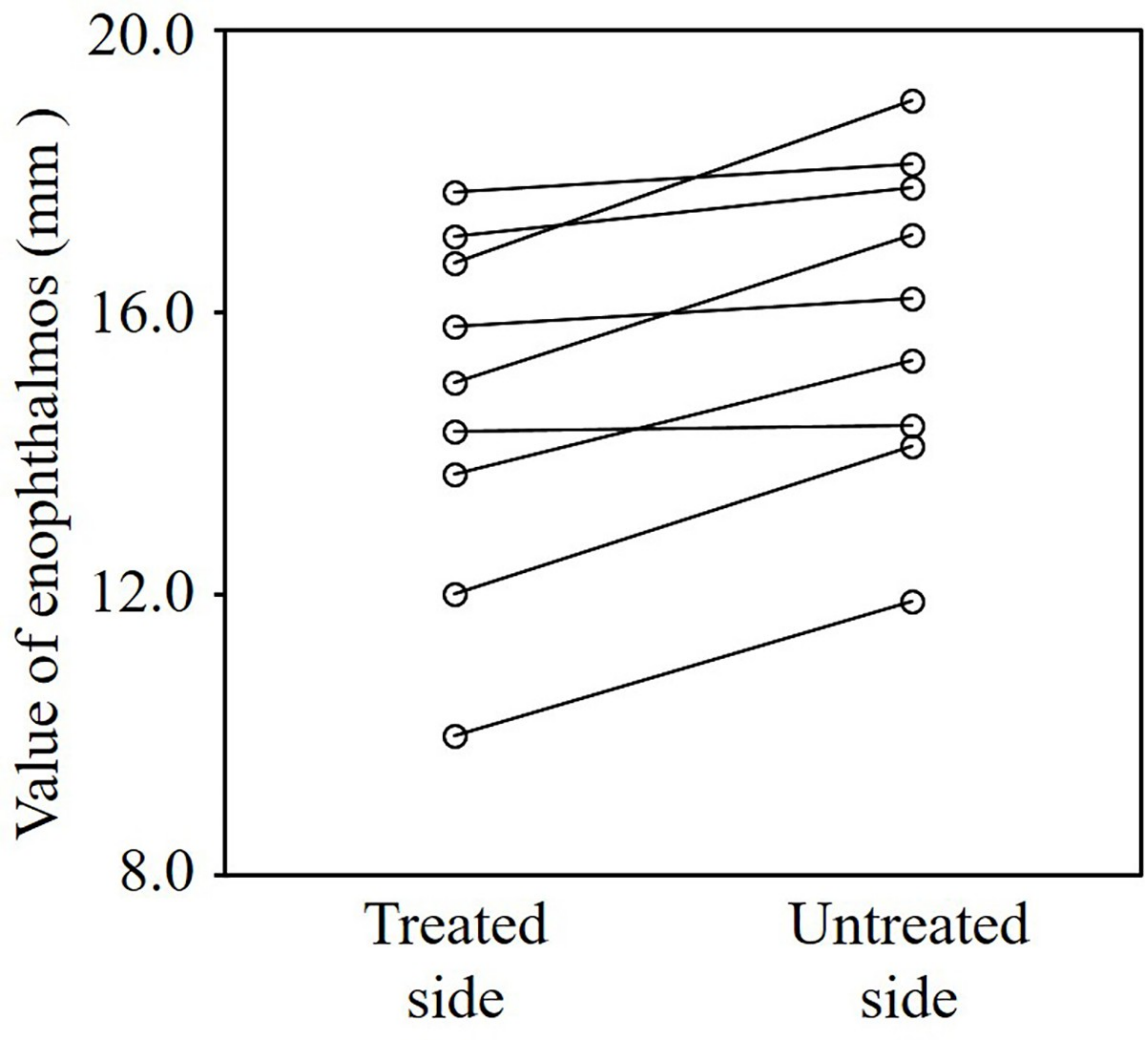
Enophthalmos values on the treated and untreated sides. The mean value on the treated side was significantly smaller than that on the untreated side (*P* = 0.002), although the differences were slight in some patients.

The mean enophthalmos values were 14.7 ± 2.5 and 16.0 ± 2.3 mm on the treated and untreated sides, respectively (Table 2). The mean value on the treated side was significantly smaller than that on the untreated side (*P* = 0.002) (Fig 5). In all patients, the enophthalmos value was smaller on the treated side than on the untreated side, although the differences were minimal in some patients.

### Relation between the orbital fat volume and enophthalmos value

**Fig 6:**
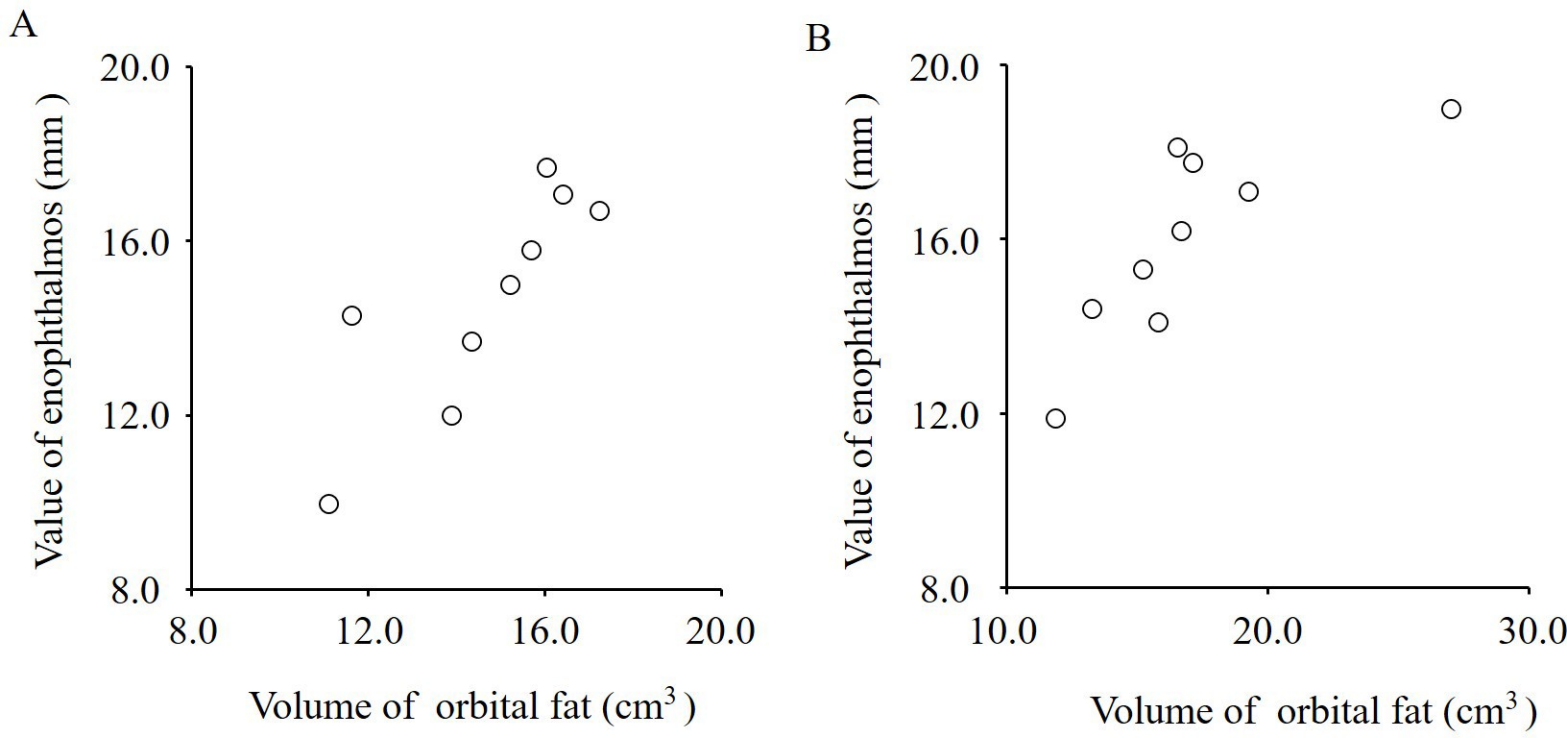
Correlation between the orbital fat volumes and the enophthalmos values. **A**, Treated side. **B**, Untreated side. The orbital fat volumes showed statistically significantly positive correlations with the enophthalmos values on both the treated (r = 0.83, *P* = 0.006) and untreated (r = 0.79, *P* = 0.01) sides.

The orbital fat volumes showed statistically significantly positive correlations with the enophthalmos values on both the treated side (r = 0.83, *P* = 0.006) and the untreated side (r = 0.79, *P* = 0.01) (Fig 6).

## Discussion

The mean volume of orbital fat and the mean enophthalmos value on the treated side were significantly smaller than those on the untreated side, whereas there were no significant differences in the mean orbital fat volumes of the other measured tissues (i.e., extraocular muscles, eyeball, optic nerve). Hence, this study quantitatively revealed that enophthalmos could be caused by the bimatoprost-induced decrease in orbital fat. In addition, the orbital fat volumes showed statistically significant positive correlations with the enophthalmos values on both the treated and untreated sides. These correlations support our hypothesized mechanism. Jayaprakasam and Ghazi-Nouri reported the MRI features of orbital fat atrophy in one patient using bimatoprost [25], but they did not show quantitatively that the decrease in orbital fat was due to bimatoprost. Our current study quantitatively revealed not only the decrease in orbital fat but also its relation to enophthalmos.

The mechanism of PAP (e.g., enophthalmos) due to PGF_2α_ analogs remains unclear. The important regulators of fat cell lipolysis are hormones—mainly insulin, catecholamines, natriuretic peptides—and paracrine factors such as prostaglandins, cytokines, and adenosine [26]. Some reports stated that activation of PGF_2α_ receptors could inhibit adipocyte differentiation [27–29]. Serrero and Lepak investigated the effect of receptor agonists on the differentiation of newborn rat adipocytes in primary cultures and reported that PGF_2α_ receptor agonists were indeed potent inhibitors of adipocyte differentiation [28]. Thus, PAPs due to PGF_2α_ analogs could be caused by that mechanism.

Enophthalmos values were smaller on the treated side than on the untreated side in all patients, and the mean enophthalmos value of the treated eyes was statistically significantly smaller than that in their contralateral eyes. A previous study investigated the frequency of enophthalmos due to bimatoprost using Hertel exophthalmometry measurements [22]. They found that a difference of ≥2 mm between the two eyes was indicative of enophthalmos, which was seen in 80% of cases. However, the accuracy of Hertel exophthalmometric measurements is questionable because the value is determined visually by the examiner. In addition, these measurements could be biased because patients treated with bimatoprost may have periorbital changes, such as pigmentation of the eyelid and iris, increased eyelash growth, and conjunctival hyphemia [10, 11, 14–19, 30]. In contrast, in the current study, the enophthalmos was measured on MRI axial images in a masked fashion, thereby minimizing measurement errors by avoiding possible bias, resulting in more accurate measurements than those obtained using Hertel exophthalmometry.

The volumes of the orbital tissues in the present study were measured using MRI. Tian et al. also reported orbital tissues volumes in 21 normal subjects [31] that were measured using MRI in a manner similar to that described herein. Their mean eyeball volume was 9.047 cm^3^, which is well matched by the mean volume we found.

The present study has some limitations. First, the study group was small, with only nine patients. The results, however, showed a similar tendency in all patients—that is, the orbital fat volumes and exophthalmos values were smaller on the treated side than on the untreated side in all patients. Unfortunately, we were unable to perform a subgroup analysis because of the small sample size. Thus, it will be necessary to confirm the study findings using a subgroup analysis and prediction modeling in a future study with a larger sample size. Second, the study was designed to be an observational, cross-sectional trial. Thus, the bimatoprost-induced decrease in orbital fat over time must be confirmed in future studies. Third, this study did not investigate whether the enophthalmos continued to recover after stopping bimatoprost. Sakata et al. reported recovery from DUES after switching from bimatoprost to latanoprost [12].At 2 months after switching analogs, the DUES had either decreased or disappeared in 11 of their 13 patients. It is also necessary in future MRI studies to confirm the recovery from decreased orbital fat and enophthalmos after stopping bimatoprost or switching from bimatoprost to another PGF_2α_ analog.

In conclusion, using MRI, we quantitatively showed that bimatoprost lowered the orbital fat volume and reduced the enophthalmos value. The enophthalmos could be caused by the bimatoprost-induced decrease in orbital fat.

## Supporting information

S1 Table Specific dataset for all individuals. (XLSX)
